# Dumbbell-Shaped Ho-Doped Fiber Laser Mode-Locked by Polymer-Free Single-Walled Carbon Nanotubes Saturable Absorber

**DOI:** 10.3390/nano13101581

**Published:** 2023-05-09

**Authors:** Serafima A. Filatova, Vladimir A. Kamynin, Yuriy G. Gladush, Dmitry V. Krasnikov, Albert G. Nasibulin, Vladimir B. Tsvetkov

**Affiliations:** 1 Prokhorov General Physics Institute of the Russian Academy of Sciences, 38 Vavilov Str., 119991 Moscow, Russia; kamynin@kapella.gpi.ru (V.A.K.);; 2 Center for Photonic Science and Engineering, Skolkovo Institute of Science and Technology, 3 Nobel Str., 121205 Moscow, Russia

**Keywords:** holmium-doped fiber, fiber laser, mode-locking, ultrashort pulses, polymer-free single-walled carbon nanotubes, dumbbell-shaped cavity, ring cavity, soliton

## Abstract

We propose a simple dumbbell-shaped scheme of a Holmium-doped fiber laser incorporating a minimum number of optical elements. Mode-locking regimes were realized with the help of polymer-free single-walled carbon nanotubes (SWCNTs) synthesized using an aerosol (floating catalyst) CVD method. We show that such a laser scheme is structurally simple and more efficient than a conventional one using a ring cavity and a similar set of optical elements. In addition, we investigated the effect of SWCNT film transmittance, defined by the number of 40 nm SWCNT layers on the laser’s performance: operating regimes, stability, and self-starting. We found that three SWCNT layers with an initial transmittance of about 40% allow stable self-starting soliton mode-locking at a wavelength of 2076 nm with a single pulse energy of 0.6 nJ and a signal-to-noise ratio of more than 60 dB to be achieved.

## 1. Introduction

In recent years, ultrashort pulse (USP) lasers have been used in many fields of science and technology because of their high peak power, stability, and scalability. Special attention is given to the USP lasers operating in the spectral range of 2–3.5 µm since such sources have a number of promising applications [1,2], including spectroscopy [3], comb-generators [4], materials processing [5], laser surgery [6,7], biodiagnostics [8], gas sensing [9], and free-space communication [10]. Compact all-fiber USP lasers, which can be the basic part of mobile sources of broadband pulsed radiation, and Raman soliton generators, as well as medical systems, are of particular interest.

Nowadays, most of the publications related to USP sources of the 2 µm spectral range discuss mode-locked thulium-doped (Tm^3+^) fiber lasers [11,12,13]. However, holmium-doped (Ho^3+^) fibers have attracted great interest due to their capability to emit in the spectral range of 2–2.2 μm, which is beyond the gain spectrum of Tm-doped materials [14]. The publication activity in this field has been constantly growing during the last 7 years. In most of the presented works devoted to pulsed Ho-doped fiber lasers operating in the solitonic regime, the passive mode-locking has been realized using material or artificial saturable absorbers (SAs) [15]. The commonly used material SAs for Ho-doped fiber lasers are carbon nanotubes (CNT) [16,17], black phosphorus [18], graphene [19], and semiconductor saturable absorber mirrors (SESAM) [20,21]. In these publications, the laser emissions demonstrate the following range of parameters: pulse durations in the range of 0.7–1.6 ps and pulse energies from 0.37 to 2.8 nJ. In [20], the authors presented the stable 37th-order harmonic mode-locked solitons with a maximum repetition rate up to 570 MHz at 2053 nm in an all-polarization maintaining Ho-doped fiber laser. Operation in the stretched-pulse dispersion regime was also demonstrated [22]. Nonlinear polarization evolution (NPE) based on the nonlinear optical Kerr effect in fibers [23,24,25,26] has been employed in a Ho-doped fiber laser as artificial SAs. Moreover, hybrid mode-locking based on combining the two types of absorbers (NPE and single-walled carbon nanotubes (SWCNT)) in an all-fiber Ho-doped cavity has been demonstrated [27,28].

In practice, each of the presented laser schemes contain such optical elements as isolators (in the ring cavity), hybrid mirrors (in the linear cavity), polarizers, beam splitters, band-pass filters, circulators, bulky elements, etc.—i.e., the components leading to additional insertion losses in the range of 13–26%. Obviously, for the further development of ultrashort pulsed lasers in complex systems or in the case of their application as an independent system, it is necessary to simplify the laser scheme and to reduce the number of elements that can affect laser generation. In addition, a decrease in the price and the service cost of such a laser is expected. One of the ways to solve this problem is to use a quite simple linear-like scheme with the so-called dumbbell-shaped cavity. These cavities consist of a fiber amplifier, standard couplers forming fiber loop mirrors, polarization controllers, and saturable absorbers if necessary [29]. Using a minimum set of optical components in the laser sources makes them more accessible. In this cavity configuration, unlike the ring and figure-eight cavities, there is no need to use an optical isolator to ensure the unidirectional propagation of radiation. Thus, the scheme is simplified, and there are no possible additional losses. Such cavity schemes have been implemented for ytterbium (Yb) [29,30], erbium (Er) [31], thulium [32], and holmium-doped [33] fiber lasers. In the last case, the laser generated only noise-like pulses with energy up to 280 nJ.

This work proposes a simplification of the mode-locked Ho-doped fiber laser scheme by using a dumbbell-shaped cavity configuration and reducing the fiber optic elements in the scheme. We designed an all-fiber holmium laser emitting ultrashort soliton pulses beyond 2 μm. Mode-locking was realized with a polymer-free SWCNT saturable absorber, which provides stable long-term pulsed generation due to its better thermal stability. We experimentally studied the effect of different SWCNTs’ film thickness (initial transmittance of SAs), placed in the cavity, on operating regimes, stability, and the self-starting of a Ho-doped fiber laser. We also compared the pulse generation parameters in the dumbbell-shaped and ring cavities of a Ho-doped fiber laser with a close set of fiber optic elements.

## 2. Experimental Setup and Materials

**Figure 1:**
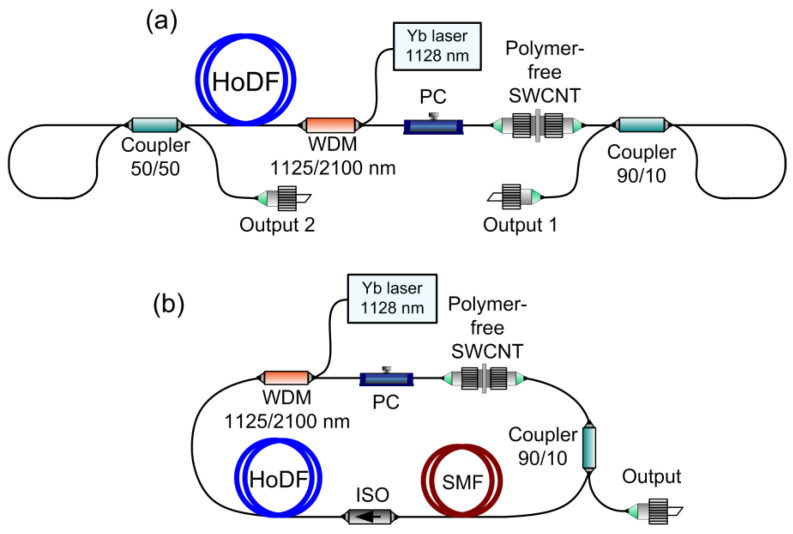
Experimental setup of the mode-locked Ho-doped fiber laser: (**a**) with a dumbbell-shaped cavity, (**b**) with a ring cavity. PC—polarization controller, HoDF—holmium-doped fiber, WDM—wavelength division multiplexer, SWCNT—single-walled carbon nanotubes, ISO—fiber isolator, SMF—single-mode fiber.

We have realized two laser schemes, shown in Figure 1. These schemes were used to study the generation parameters of a Ho-doped fiber laser with a dumbbell-shaped cavity and to compare the laser generation parameters of two cavity types with a close set of elements [34]. The laser cavities include 2 m of Ho-doped fiber with counter-propagating pumping realized using a continuous-wave (CW) Yb-doped fiber laser at a wavelength of 1128 nm through a 1125/2100 nm wavelength division multiplexer (WDM). The maximum losses at the used WDM were up to 15% (pump port–common port). The pump wavelength was determined using the holmium ions absorption spectrum with a ^5^I_8_ → ^5^I_6_ transition (corresponded to a wavelength of 1150 nm), as well as with the spectral dependence of the Yb-doped fiber laser’s efficiency, which decreases at wavelengths more than 1120 nm [35]. Therefore, we choose a wavelength of the Yb-doped fiber laser not exceeding 1130 nm for most experiments. It ensures a sufficiently high generation efficiency and has a high absorption of holmium ions. The power of the Yb-doped fiber laser was up to 4 W. The pair of standard couplers with 50/50 and 90/10 coupling ratios forming fiber loop mirrors were used as the high (≈99%) and low (≈40%) reflective mirrors, respectively [36], in a dumbbell-shaped cavity (Figure 1a). The 90% port of a fiber coupler with 90/10 coupling ratios outcoupled the radiation of the Ho-doped fiber laser with the ring cavity (Figure 1b). Losses on the fiber couplers were in the range of 15–20% (the difference between the input port power and the total power from both output ports). The saturable absorber specimens were fixed between two standard angle-polished optical connectors (FC/APC). The intracavity polarization states were tuned using the squeezer-based polarization controller (PC) placed between the WDM and the saturable absorber. To select one propagation direction of the laser radiation in the ring cavity, we used a fiber isolator (ISO) operating at wavelengths above 2 μm for which the insertion losses did not exceed 20%. All of the fibers and optical fiber components used were commercially available and based on silica fiber, which simplified the implementation of the splicing’s with the lowest possible losses on them. Angle-polished optical connectors were used at the output of the lasers to prevent back reflection of the radiation. In the dumbbell-shaped cavity, the laser radiation characteristics were measured at output 1. At output 2, we could observe the residual unabsorbed pump from the Yb-doped fiber laser and a weak generation signal similar to the emission at output 1.

We kept the same pulse repetition rate in the different types of laser cavities during the comparison of the laser radiation parameters. The pulse repetition rate depends on the cavity length. As was mentioned above, the dumbbell-shaped cavity is a linear type scheme. It follows that the lengths of the presented and ring cavities will be differed by a factor of two for the same repetition rate in the mode-locking regime in the case of the same cavity media. The total length of the dumbbell-shaped cavity was about 8.7 m, which corresponded to the pulse repetition rate of about 12 MHz. The total length of the ring cavity was about 17.4 m, achieved by adding a section of about 9.5 m of single-mode fiber (SMF) to obtain the same repetition rate.

The Ho-doped fiber as a gain medium has an absorption of ~5.5 dB/m at a pump wavelength of 1128 nm and a group-velocity dispersion of *β_2_* ≈ −0.112 ps^2^/m around 2100 nm. The fiber core diameter was 13 μm and the holmium ions concentration in the active core was estimated as 2 × 10^19^ cm^−3^. In addition to the Ho-doped active fiber, the laser cavities were formed using a standard single-mode fiber SMF-28. So, the net group velocity dispersion of the dumbbell-shaped cavity was estimated as −0.9 ps^2^, and for the ring cavity, as −1.8 ps^2^, which contributes to the soliton operation mode of both lasers.

**Figure 2:**
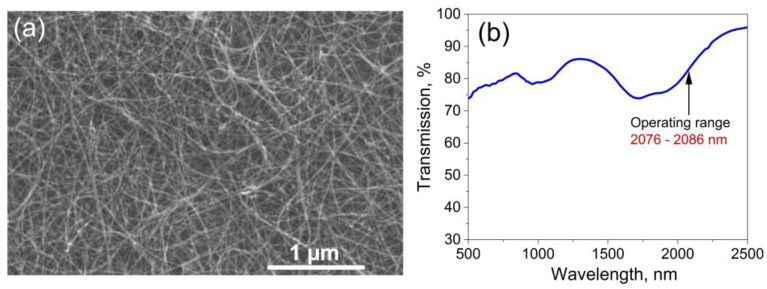
(**a**) Scanning electron microscopy image of the SWCNT film with 40 nm thickness, scale bar represents 1 µm; (**b**) transmission spectrum of the used polymer-free SWCNT film.

We have used polymer-free SWCNT films as a saturable absorber fixed between two FC/APC fiber connector end facets [37]. This type of nanotube has been successfully used to obtain mode-locking in bismuth [38], Er [39] and Tm-doped [40,41] fiber lasers. In the last case, dual-wavelength soliton generation was realized [41]. Using such an absorber type for mode-locking is very promising since it could avoid the thermal degradation problem of conventional CNTs dispersed in a polymer matrix film [42]. The SWCNTs were synthesized using the aerosol (floating catalyst) chemical vapor deposition technique [43]. The SWCNTs were grown on the surface of iron-based catalyst particles via the Boudouard reaction. The catalyst was suspended (in the aerosol phase) in the CO atmosphere. The synthesis parameters (namely, CO_2_ promoter gas concentration and temperature) were tuned so that the mean diameter corresponded to the optical transition around 1700–1900 nm [44]. The material was collected on a nitrocellulose filter. Figure 2a shows a scanning electron microscopy (SEM) image of the used SWCNT film. The thickness of the SWCNT film was controlled using the collection time and was around 40 nm for this experiment, corresponding to about 83% transmittance in the operating spectral range of 2076–2086 nm (Figure 2b). The SWCNT films were transferred from the filter to the desired substrate or on the end of the FC/APC connector directly using a simple dry transfer technique [45]. This process does not require any liquid dispersion and purification steps, and the film does not use any polymer support.

In addition to simplifying the laser scheme, we investigated the stability and repeatability of the laser’s operation. For the Ho-doped fiber laser with a dumbbell-shaped cavity, we studied the effect of the number of SWCNT film layers (1, 2, 3, and 6) placed inside the cavity on the laser operating regimes, stability, and self-starting. The measurement of the nonlinear optical transmittance of different SWCNT film layers using the energy-dependent transmission technique was carried out. A homemade Ho-doped fiber laser generating 1.3 ps pulsed radiation with a repetition rate of about 12 MHz at a central wavelength of 2076 nm was used as the probe laser source. The Ho-doped fiber laser output parameters were fixed during the measurement. The application of a variable optical attenuator allowed us to vary the laser’s radiation energy from 0 up to 300 pJ, supplied to both arms (reference and test) and formed by a 50/50 fiber coupler. The test arm contained SWCNT film samples fixed between two FC/APC connectors. We determined the nonlinear saturable absorption of the samples under study by comparing the pulse energy values at the outputs of the reference and test arms.

**Figure 3:**
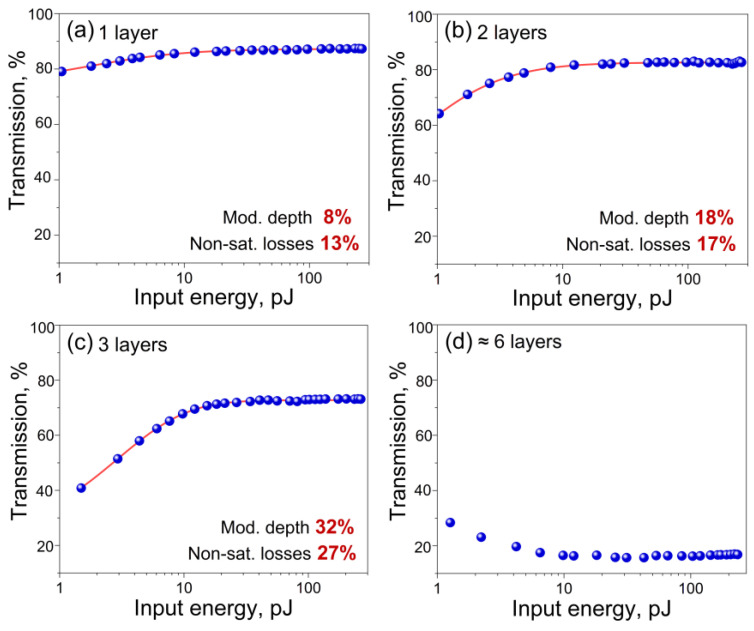
Energy-dependent transmittance of different SWCNT samples under the excitation of 1.3 ps pulses at 2076 nm: (**a**) 1 layer, (**b**) 2 layers, (**c**) layers, (**d**) ≈6 layers. Circles—experimental data, red curve—theoretical fit.

Figure 3 shows the measured nonlinear transmission of various SWCNT film layers. The experimental data were approximated with a fast saturable absorber model as in [46]. The calculated modulation depths and non-saturable losses for various SWCNT film samples are indicated on the corresponding graphs in Figure 3. We did not observe the SWCNTs’ degradation during and after the nonlinear optical transmittance measurements in the whole incident energies range for all samples. With the increase in the number of layers, an increase in the values of modulation depth and non-saturable losses was observed. The one-layer SWCNT film transmission measured under low-intensity exposure is in good agreement with the obtained linear transmission spectrum (Figure 2b). For six layers of SWCNT films (Figure 3d), another character of the nonlinear transmission distribution was observed. It can be associated with the thermal destruction of a relatively thick total film layer under the action of laser radiation.

The laser and SWCNT films characteristics were studied using the following equipment: an optical spectrum analyzer (Avesta ASP-IR-2.6), an oscilloscope Tektronix MSO 64 (4 GHz) coupled with a 5 GHz photodiode IBSG PD24 (0.8–2.4 µm), a radio frequency (RF) spectrum analyzer with a 26.5 GHz bandwidth (Keysight 9020B), and a scanning autocorrelator (Avesta AA-10DD-30ps).

## 3. Experimental Results and Discussion

We have studied the effect of the number of SWCNT film layers (1, 2, 3, and 6) placed inside a dumbbell-shaped cavity on the output characteristics of a Ho-doped fiber laser. When a single 40 nm SWCNT film is used in the laser cavity, unstable mode-locking at a wavelength of 2086 nm is observed at a pump power of 0.83 W, which breaks down into the CW operation mode. As the pump power was increased up to 0.95 W, unstable harmonic mode-locking with a doubling of the pulse repetition rate (24 MHz) was observed. In this case, the average output power was 18.2 mW. However, with a fixed position of the polarization controllers, self-starting of the laser was not observed.

**Figure 4:**
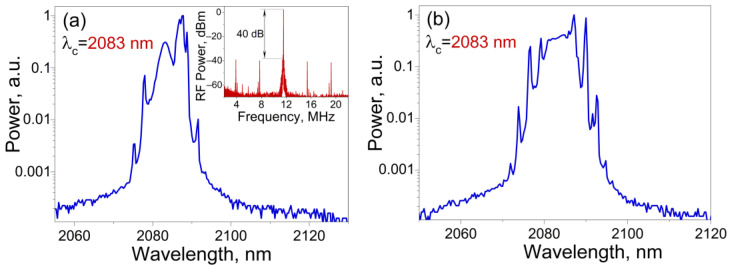
Output optical spectra of a Ho-doped fiber laser with two layers of SWCNTs in the dumbbell-shaped cavity: (**a**) with a CW component at Pp = 0.9 W, RF-spectrum on the inset; (**b**) spectrum corresponding to a disordered sequence of pulses at Pp = 1 W.

For the case of two SWCNT film layers, mode-locking at a wavelength of 2083 nm was realized at a pump power of 0.9 W. The average output power was 9.4 mW at the fundamental pulse repetition rate of 11.5 MHz. This is due to the longer cavity length, since the fiber section in the bundle of optical FC/APC connectors with fixed SWCNT films, was slightly longer than the previous one. The pulse duration was 1.5 ps and slightly chirped (TBP = 0.321). However, during long-term operation, a CW component was observed in the optical spectrum (Figure 4a). In addition, besides the main peak, additional peaks were observed in the radio frequency (RF) spectrum, so the signal-to-noise ratio was only 40 dB (the inset in Figure 4a). As the pump power was increased up to 1 W, we observed a disordered sequence of pulses with the corresponding spectrum shown in Figure 4b. In this case, as well as for the one layer, we failed to achieve self-starting of the laser system.

**Figure 5:**
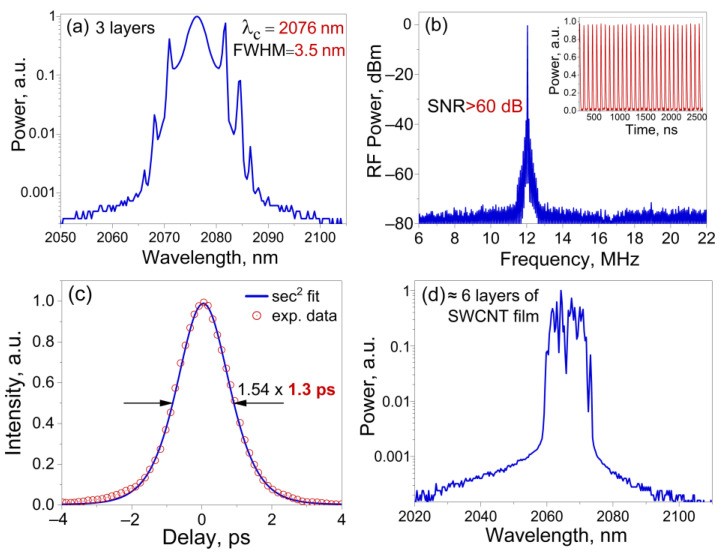
Output characteristics of a Ho-doped fiber laser with three layers of SWCNTs in the dumbbell-shaped cavity: (**a**) optical spectrum; (**b**) radio frequency spectrum, and oscilloscope trace on the inset; (**c**) pulse autocorrelation; (**d**) the output emission spectrum with six layers of SWCNT films in the laser cavity at Pp = 2 W.

Stable single-pulse self-starting mode-locking was observed at a wavelength of 2076 nm and a fundamental repetition rate of 12 MHz with three layers of SWCNT films in the laser cavity (Figure 5a–c). The blue shift of the central wavelength observed in all optical spectra may be due to the increasing non-saturable losses of SWCNT films. The pulse repetition rate precisely matched the laser cavity roundtrip. The lasing threshold was at a pump power of 0.75 W, at which a CW lasing was observed. As the pump power was increased above 0.8 W, the laser turned to a transient unstable Q-switching state and then transformed into a mode-locking operation after minimal PC adjusting. The average output power was 7 mW at a pump power of 0.95 W, corresponding to the single pulse energy of 0.6 nJ. The RF spectrum had a signal-to-noise ratio of more than 60 dB (Figure 5b). The measured autocorrelation trace of soliton with a 1.3 ps pulse duration is presented in Figure 5c with a sech^2^ fitting. It should be noted that, in this case, at the first launch, the laser operated in the required mode with minimal adjustments to the PC. During subsequent starts at a fixed pump power and optimal position of the polarization controller, chaotic generation of an unstable Q-switching producing stochastic energetic nanosecond pulses was observed, which then turned into stable mode-locking after 10–30 s.

With an increase in the number of layers of SWCNT films, their uniform application procedure to the end face of the FC/APC connector becomes more complicated. We investigated the laser’s characteristics with six layers of SWCNT films in the cavity and we observed neither mode-locking nor Q-switching or CW generation. The lasing threshold increased up to 2 W and the average output power was approximately 11 mW. The output emission spectrum at these values is shown in Figure 5d. This can be explained by the fact that the too high unsaturated absorption resulted in thermal damage to the SWCNT films. So, this means that the given number of SWCNT layers introduces overly large losses into the laser cavity and this is unacceptable for mode-locking achievement. Thus, based on the obtained results, we believe that repeatability of the laser generation parameters is possible for a small number of SWCNT film layers, namely for three or two layers.

We also compared the generation parameters in two types of Ho-doped fiber laser cavities with a similar set of fiber optic elements. To compare the dumbbell-shaped and ring cavity generation parameters under similar conditions, we kept the same pulse repetition rate of about 12 MHz and used the same three layers of SWCNT film fixed between the FC/APC connectors in the ring cavity to obtain mode-locking. The lasing threshold was observed at a pump power of 2 W, and a mode-locking regime at 2.2 W after PC adjustment. Thus, we obtained pulsed radiation with a central wavelength of 2050 nm, the average output power of about 12 mW, and a pulse duration of 1.5 ps with 1 nJ of energy. We did not observe self-starting of the laser system. So, at similar generation parameters, the ring cavity of a Ho-doped fiber laser requires twice the pump power.

As is well-known, in the case of ultrashort pulse generation in a medium with anomalous dispersion, the formation of a soliton-like pulses occurs, which we observed in both cases of the different cavity configurations. In this case, one can observe characteristic Kelly sidebands on the optical spectra (Figure 5a). However, the most significant difference between the dumbbell-shaped cavity scheme and the ring one is the double pass of the generated pulses through both the amplifier medium (Ho-doped fiber) and the saturable absorber (SWCNT). Table 1 summarizes the obtained laser generation characteristics depending on the different number of SWCNT layers used in the dumbbell-shaped cavity, as well as a function of the cavity type.

Table 1 shows that stable mode-locking regimes can be obtained using two or three layers of SWCNT film applied to the ends of the FC/APC optical connectors. In all cases, a soliton generation mode with minimal pulse frequency modulation was realized. However, self-starting was observed only for the dumbbell-shaped cavity type with three layers of SWCNT film. Changing the cavity configuration to the ring one with a similar set of optical elements leads to a 2-fold increase in the pumping value required to implement stable mode-locking, and also leads to a shift in the lasing wavelength to the short-wavelength range.

Thus, taking into account the required pump power level and the progress in the field of laser technologies, it will be possible to pump the Ho-doped fiber laser with an optimized dumbbell-shaped cavity directly using a semiconductor laser diode in the wavelength range of 1125–1150 nm [47]. This will allow the transition to more technologically advanced, simple, and compact pulsed Ho-doped fiber laser systems, as well as expand the possibilities for their application.

## 4. Conclusions

To conclude, we have demonstrated the first implementation of soliton pulses in a Holmium-doped fiber laser with a dumbbell-shaped cavity and using aerosol-synthesized polymer-free SWCNT films as a saturable absorber. Using such an absorber type for mode-locking in this cavity configuration allowed us to avoid the degradation problem of conventional CNTs dispersed in a polymer matrix film. We experimentally studied the effect of 1, 2, 3, and 6 SWCNT film layers (40 nm for each layer) placed in the cavity on the operating regimes, stability, and self-starting of the laser. For all samples of SWCNT films, the energy-dependent measurement of the nonlinear optical transmittance was carried out. Using single or double SWCNT film layers led to unstable breakdown mode-locking. In turn, three layers of polymer-free SWCNT films with a small signal transmittance of about 40% allowed one to obtain stable self-starting soliton mode-locking at a wavelength of 2076 nm with a pulse duration of 1.3 ps and a good signal-to-noise ratio of more than 60 dB. The average output power was 7 mW, corresponding to the single pulse energy of 0.6 nJ.

Thus, we have shown that a Ho-doped fiber laser with a dumbbell-shaped cavity and a minimum set of optical elements is capable of generating solitons and provides a competitive alternative to a mode-locked laser with a conventional ring cavity configuration. Compared to the ring laser cavity, the dumbbell-shaped laser cavity is structurally simple and more efficient. We believe that such a cavity configuration in combination with appropriate absorbers has promise for subsequent use in scientific and practical tasks.

## Figures and Tables

**Table 1:** The obtained characteristics of laser radiation as a function of the different amount of 40 nm SWCNT film layers used in the dumbbell-shaped cavity of a Ho-doped fiber laser, and as a function of the laser cavity type. D—dumbbell-shaped cavity, R—ring cavity, α_0_—estimated modulation depth, nGVD—net group velocity dispersion of the cavity, λc—central wavelength, FWHM—optical spectrum bandwidth at half maximum, Frep—pulse repetition rate, Pp—pump power, Pavg—average output power, τ—pulse duration, E—pulse energy, TBP—time-bandwidth product.

Cavity Type	Number of SWCNT Layers	α_0_ [%]	nGVD [ps^2^]	λc [nm]	FWHM [nm]	Frep [MHz]	Pp [W]	Pavg [mW]	τ [ps]	E [nJ]	TBP	Self- Start
D	1	8	−0.9	2086	3.0	12.0	0.83	10.5	CW + ML (1.6)	0.9	0.331	No
D	2	18	−0.9	2083	3.0	11.5	0.9	9.4	1.55	0.8	0.321	No
D	3	32	−0.9	2076	3.5	12.0	0.95	7.0	1.30	0.6	0.317	Yes
R	3	32	−1.8	2050	3	12.02	2.2	12	1.5	1	0.321	No

## Data Availability

The data supporting the findings of this study are available within the article.
